# Book review: The first sense

**DOI:** 10.3389/fpsyg.2015.01196

**Published:** 2015-08-11

**Authors:** Tony Cheng

**Affiliations:** Department of Philosophy, University College LondonLondon, UK

**Keywords:** touch, exploratory action, multisensory integration, unity of touch

## Introduction

*The First Sense: A Philosophical Study of Human Touch* is one of the rare contributions in theoretical and philosophical psychology exclusively on human's sense of touch in the past decades. Although the study is conducted from a philosophical point of view, it is highly empirically informed. The author seeks to base his distinctions and arguments on empirical findings, but also offers his own original ideas and theses. In Section The Structure and Contents of the Book I discuss the structure and contents of the book with an emphasis on the central thesis “the unity of touch,” and in Section A Different Perspective I contrast that central thesis with a different perspective.

## The structure and contents of the book

The book is divided into seven chapters, starting from the preliminary chapter “what is touch?” This opening chapter lists varieties of examples of human touch, and draws some important distinctions, such as cutaneous (i.e., “those which are processed solely by the sensory receptors located in the skin,” Fulkerson, 2014, p. 6) vs. haptic (i.e., those which “[involve] a combination of cutaneous stimulation along with kinesthetic and other motor feedback,” Fulkerson, 2014, p. 6), body directed versus object directed, and so on. Chapter 2 is the central chapter of the book, arguing that even though many recent works have shown that touch is multisensory in one way or another, “none of the truly interesting ways of characterizing multisensory experiences can be used to show that touch experiences are *uniquely* multisensory” (Fulkerson, 2014, p. 18, my emphasis). The idea is that by whatever standard touch is multisensory, other senses such as vision and audition would be multisensory too, so these standards do not refute the idea that touch is an unitary sense modality. Chapter 3 discusses exploratory action in touch (i.e., active, haptic touch), in particular feature binding, grouping, segmentation, and so on. This chapter is important not only in its own right, but also in relation to the previous chapter, as the key argument the author puts forward in supporting the unity of human touch thesis hinges on the notion of exploratory action. Chapter 4 evaluates the complex relations between touch and bodily awareness. In particular, there have been several dependency relations put forward by other theories, including causal, inferential, experiential dependence, among others. The basic idea is that touch is in some way dependent upon bodily awareness (e.g., Martin, 1992; Noë, 2004). After surveying others' views, the author goes on to argue for one version of this dependence view. Chapter 5 discusses varieties of tangible qualities. This topic is important for many reasons, one being that Aristotle (1941) proposed an important idea that the way to individuate the senses is to use the idea of “proper sensibles,” for example colors for vision, sounds for audition, and so on. This view is in trouble when it comes to touch, as Aristotle himself acknowledged, since unlike vision, audition, olfaction and gustation, “in the field of what is tangible we find several such pairs, hot cold, dry moist, hard soft, etc.” (Aristotle, 1941, book 2, chapter 11). The author's view is that “intrinsic geometrical properties available to haptic touch is not “basic,” in the sense that “[they] are not maximal determinates of some determinable, nor are they maximally extracted object features” (Fulkerson, 2014, p. 121). Chapter 6 explores the possibilities of distal touch, e.g., tactile filling in, volume touch, indirect touch, and tactual projection. The targets being criticized are versions of “contact thesis,” which holds that “[tactual] object perception occurs only at the surface or limit of the body” (Fulkerson, 2014, p. 142), or the weaker view that *apparent* surface or limit is in play. The author argues for a different view that “[tactual] reference to an object requires an appropriate tactual connection with the object, either directly or through some intermediary” (Fulkerson, 2014, p. 147, the “connection principle”). Chapter 7 discusses pleasant touch, exploring the affective and emotional aspects of touch. The book is well structured with substantial contents.

The author has covered a wide range of issues in this book, so some readers might wonder about the author's comprehensive picture of tactile sensory system. Here is my understanding: the author agrees that physiologically speaking, there are several distinct informational channels when it comes to touch. However, when one attempts to provide explicit criteria (functional dissociation, shared content, multiple stimulus) for the idea that touch is *not* one unified sensory modality, the author argues that none of them is defensible. Now, what about the relations between this central thesis and other ideas defended in other chapters? It is certainly *not* the case that most other ideas hinge on the plausibility of the unity thesis. However, some of those other ideas do have significant role to play in supporting the unity thesis, for example the discussion of explorative action and haptic touch in chapter 3. In general, even if the unity thesis fails, many of the author's other views might still be defensible, for example it is not obvious that the author's discussion of pleasant touch will rise or fall with the unity thesis. Now let's have another look at the central thesis of the book and contrast it with a different perspective.

## A different perspective

The author in chapter 2 argues for the “unisensory view” and against the “multisensory view” (Fulkerson, 2014, p. 18). The basic idea is this:

In haptic touch, the various cutaneous and kinesthetic activations are coordinated…through exploratory action, resulting in a unified perceptual experience of tangible objects. The unified representations that result are structurally similar to those found in vision and the other senses and can be contrasted with the kind of representations typical of multisensory experiences. Haptic touch thus turns out to be a single modality, its various constituent systems aligned much like those involved in vision, audition, and the other senses. (p. 18).

This view is hard to defend in light of many recent works on multisensory integration (e.g., Jones and Lederman, 2006), but the author encounters the challenge directly and makes the unisensory view much more plausible than it normally seems. This short review cannot go into the details of this debate, but below I briefly introduce a different perspective to highlight the focal disagreement.

In arguing for the unisensory view the author focuses on *haptic* touch, but in many experimental paradigms that seek to tease tactile and thermal perception or tactile perception and bodily sensation apart, *cutaneous* touch is crucial. Now an alternative conception is this: while bodily sensation is interoceptive, both thermal perception and tactile perception (including haptic touch) are exteroceptive. Moreover, while thermal perception is not in general object-directed, tactile perception is. A specific development of this idea is the view that while there is a tactile field that is analogous to visual field, there is no nociceptive field (e.g., Haggard and Giovagnoli, 2011; Mancini et al., 2015). This can be a basis of the competing view that there are several senses of touch, some are exteroceptive and object-directed, some are not, for example. This view itself is no less controversial than the author's view, but it seems to be a reasonable competing picture. It would be nice to further investigate this field-based view and the author's unisensory view on future occasions.

### Conflict of interest statement

The author declares that the research was conducted in the absence of any commercial or financial relationships that could be construed as a potential conflict of interest.
